# Optimization of Cold Chain Distribution Route with Mixed Time Window considering Customer Priority

**DOI:** 10.1155/2022/2953205

**Published:** 2022-09-09

**Authors:** Shouchen Liu, Cheng Zhang

**Affiliations:** ^1^School of Business Administration, Fujian Business University, Fuzhou 350012, China; ^2^School of Transportation Engineering, East China Jiao Tong University, Nanchang 330013, China

## Abstract

In order to study the mixed time window vehicle routing optimization problem based on customer priority, a customer differentiation management strategy based on customer priority is proposed. Combined with the main factors affecting customer priority evaluation and the characteristics of vehicle routing problem with mixed time windows, a comprehensive evaluation index affecting customer priority was first established and DBSCAN clustering algorithm was used for clustering analysis of customer priority to solve the optimization problem of cold chain distribution route considering customer priority. Fuzzy time window of refrigerated vehicles was then constructed with trapezoidal fuzzy number, and a mathematical programming model was built with an objective function for minimizing the sum of fixed, green, penalty, refrigeration, and cargo damage costs. Two scenarios of out-of-stock and not-out-of-stock were designed. Finally, an improved genetic algorithm was used to solve the model, and the rationality of the model was verified through a case of imported fruit distribution in Xiamen City. Results showed that the proposed method can effectively solve the routing problem of refrigerated trucks considering customer priority. Moreover, the findings of this study can provide a new approach for solving the routing optimization problem of refrigerated trucks considering customer priority.

## 1. Introduction

China's cold chain logistics market has huge potential, with the market size reaching 339.1 billion yuan in 2019. In 2020, the market size of China's cold chain logistics industry reached 372.9 billion yuan, an increase of 33.8 billion yuan over 2019, with a year-on-year growth of 9.97%. With the continuous improvement of people's living standards and the acceleration of urbanization, people have higher safety awareness and quality requirements for food. Coupled with the continuous promotion of the Belt and Road, the trade of agricultural products along the Silk Road is carried out enthusiastically, and the demand for cold chain increases rapidly. Coupled with the steady development of fresh products and e-commerce, China's cold chain logistics will usher in opportunities for rapid development. In the complex cold chain distribution market environment, how to reduce the distribution distance of cold chain logistics, reduce logistics costs, and improve customer satisfaction has become a crucial issue.

Meeting the needs of a flourishing society is difficult with the existing cold chain logistics equipment and development level as well as the objective of building a thriving society in an all-around way. The unreasonable layout of cold chain distribution nodes and unscientific distribution route lead to high cost and low efficiency of cold chain logistics distribution. Scientific and reasonable cold chain transportation routes can reduce cost and improve the efficiency of cold chain logistics. According to Pareto rule, 20% of customers create 80% of profits of the enterprise. Hence, the enterprise should accurately locate customers, seize the few key customers, and improve the service level of these key customers. This rule also applies to cold chain logistics and distribution enterprises. Such enterprises need to capture the key 20% of customers and improve their satisfaction. Improving the cold chain distribution service level as well as customer satisfaction and loyalty can create additional value and benefit while maintaining the cold chain distribution cost in the increasingly fierce competition in the industry.

## 2. Literature Review

With the development of globalization and social networking, cold chain logistics in the context has become the focus of all countries and an important field of international cooperation. Research on vehicle routing problem of cold chain logistics mainly focuses on aspects of time window, carbon emission, consider traffic congestion, and customer satisfaction. Studies on the distribution time window mainly include: Li et al. [1] comprehensively considered the mixed integer model of multiobjective route optimization with minimum cost and time as the objective function of refrigerated vehicle traveling speed, load weight, and mileage and used NSGA-II algorithm to solve it. Tu et al. [2] utilized distance-matrix multiplier network theory to build a fresh vehicle route optimization model. Amorim et al. [3] considered perishability of cold chain agricultural products and built a multiobjective optimization model with minimum distribution cost and maximum product freshness as the objective function. Kuo [4] built a route optimization model with minimum fuel consumption as the objective function and designed a heuristic algorithm to solve the model. Tao et al. [5] established a distribution route optimization model with minimum distribution cost as the objective function and designed an artificial fish swarm algorithm to solve the model according to cold chain characteristics. However, domestic and foreign scholars have increasingly focused on carbon emissions in the distribution process and theoretically investigated the impact of low carbon and other factors on the driving route of cold chain vehicles. For example, Duan et al. [6] constructed an optimization model of agricultural cold chain logistics distribution considering carbon emissions and designed an improved artificial bee colony algorithm to solve the model. Jabir et al. [7] established the vehicle route optimization model of multi-dearage and solved the model using metaheuristic algorithm under the background of low carbon. Ge et al. [8] built a low carbon logistics distribution optimization model and analyzed the impact of three carbon emission mechanisms, namely, carbon trading, quota, and tax rate, on carbon emissions, total cost, and distribution route. Urban traffic congestion has become a normal state and travel time of road sections changes constantly with time due to the continuous growth of urban traffic load in China. Zhang et al. [9] established a cold chain distribution route optimization model given that refrigerated vehicles are distributed at different times and speeds in the urban road network. Simulated annealing algorithm was adopted to solve the model considering the scheduling problem of refrigerated vehicles for product distribution in multitemperature areas under time-varying road network environments. Kok et al. [10] built an urban refrigerated vehicle route optimization model according to characteristics of urban traffic congestion and solved the model with Tabu search algorithm. Yao et al. [11] established a distribution route optimization model considering the time-varying traffic network with the minimum total cost as the objective function. Meanwhile, domestic and foreign scholars have gradually begun to examine the importance of customer satisfaction in logistics service. The completion of delivery of cold chain products within the time window allowed by customers can remarkably improve customer satisfaction because issues of perishable cold chain products, short shelf life, and strong timeliness of distribution are addressed. In this regard, Moghaddam et al. [12] built an optimization model for refrigerated vehicle configuration and distribution route under the background of uncertain demand to improve customer satisfaction. Ren et al. [13] constructed an optimization model of cold chain distribution route based on customer satisfaction of cold chain distribution with the minimum total cost of cold chain distribution as the objective function and solved the model with an improved ant colony algorithm. Liu et al. [14] established an optimization model of urban cold chain transportation and designed an artificial immune particle swarm optimization algorithm to solve the model. Zuo et al. [15] revealed that customer satisfaction can be significantly improved by enhancing the current B2C logistics service quality. Yan et al. [16]using GIS/GPS online real-time distribution system to deliver cigarettes can save more time and logistics costs when choosing the route model of dynamic + fixed optimization. Yu et al. [17] proposed a new vehicle routing problem, called the two-level vehicle routing problem (2E-VRPTW-Co-OD), with time windows, options, and temporary drivers.

To conclude, although cold chain distribution time window, carbon emission, and customer satisfaction have been intensively investigated, the priority of customers is determined according to the information of customer order frequency (month), order amount (month average), satisfaction, loyalty, type, and level. Meanwhile, studies on vehicle routing optimization with mixed time window considering customer priority are limited.

1. Investigations typically focus on the optimization of cold chain vehicle driving route under the condition of sufficient cold chain supplies. However, studies on the urban cold chain vehicle driving route under the condition of lacking cold chain supplies are limited.
2. The cold chain transportation route problem has been extensively investigated by domestic and foreign scholars, but customer demand priority has been generally ignored.

Thus, in view of these research deficiencies, the main contributions of the article are summarized as follows:

1. The mathematical model was built under the background of a shortage of cold chain supplies, and two scenarios of shortage and no shortage were designed, which could realize the rational distribution of cold chain supplies and solve the optimal route.
2. In order to improve the distribution efficiency under the background of shortage of cold chain materials, a customer priority evaluation index is constructed to evaluate the priority of customers, and implement differentiated management for customers in the distribution process. Then, for the cold chain material distribution allocation and vehicle routing optimization decision, a cold chain logistics distribution routing optimization model with mixed time window constraints is established.
3. Based on the greedy algorithm, the crossover operator of the genetic algorithm was improved to enhance the local search ability of the algorithm, and the improved genetic algorithm was designed to solve the truck route optimization model of mixed time window. The results show that the improved genetic algorithm was superior to other intelligent algorithms in solving efficiency and searching quality.

The remainder of this article is organized as follows. The evaluation index of customer priority construction is presented and DBSCAN clustering algorithm is designed for customer clustering analysis in Section 3. An urban cold chain transport model considering customer priority is established with the minimum total cost as the objective function in Section 4. An improved genetic algorithm is designed to solve the model in Section 5. The cold chain transportation of imported fruits in Xiamen for empirical analysis and the solution of two scenarios of not-out-of-stock and out-of-stock are designed and the calculation results are discussed in Section 6. Finally, the summary of this article is presented in Section 7.

## 3. Evaluation Index and Algorithm of Customer Priority

Customer priority refers to the measurement and classification of customers by cold chain distribution enterprises from multiple perspectives according to evaluation indicators of customer priority for the classified management of customers. Cold chain distribution enterprises will give preference to customers with high priority in providing delivery services when customer demand for cold chain goods is greater than the inventory of goods.

### 3.1. Evaluation Index System of Customer Priority

According to the literature [18, 19] and demand for cold chain customer visit investigation, the demand for customers in the logistics network demonstrates multiple attributes, such as customer priority order frequency (month), order amount, satisfaction and loyalty, customer type, and customer level, such as comprehensive analysis of customer priority. Data, including relevant information of customer priority evaluation index, are obtained from the customer and order information of a cold chain logistics enterprise in Xiamen for 2020. DBSCAN clustering method is adopted in this study to analyze customer priority given a large amount of total sample data. Cold chain distribution enterprises adopt differentiated services to carry out precise distribution, strengthen customer relationship, and enhance enterprise competitiveness by recognizing the characteristics of different customers.

### 3.2. Basic Principle of the DBSCAN Algorithm

The DBSCAN clustering algorithm is a set of neighborhoods that describe the tightness of the sample set, and parameter (*ε*, MinPts) is used to describe the tightness of the sample distribution in the neighborhood [20], where *ε* refers to the neighborhood distance threshold of a certain sample and MinPts refers to the threshold of the number of samples in the neighborhood of a certain sample with a distance of *ε*. If the sample set is assumed to be *D*=(*x*_1_, *x*_2_, ..., *x*_*m*_), then the density description of the DBSCAN clustering algorithm is defined as follows:

1. *ε*− neighborhood: *ε*− neighborhood of *x*_*j*_ ∈ *D* contains subsample sets with a maximum distance of *ε* from the sample set *D*=(*x*_1_, *x*_2_, ..., *x*_*m*_) to the sample set *x*_*j*_, that is, the number of subsample set *N*_*ε*_(*x*_*j*_)={*x*_*i*_ ∈ *D*|distance(*xi*, *xj*) ≤ *ε*} is denoted |*N*_*ε*_(*x*_*j*_)|.
2. Core object: if *N*_*ε*_(*x*_*j*_) corresponding to the *ε*− neighborhood of *x*_*j*_ ∈ *D* contains at least MinPts samples and |*N*_*ε*_(*x*_*j*_)| ≥ MinPts, then *x*_*j*_ is the core object.
3. Density direct: if *x*_*i*_ is in the *ε*− neighborhood of *x*_*j*_ and *x*_*j*_ is the core object, then *x*_*i*_ can be denser direct to *x*_*j*_. However, the converse is not necessarily true, that is, *x*_*j*_ is not automatically as dense as *x*_*i*_ unless *x*_*i*_ is also the core object.
4. Density reachable: if the sample sequence *p*_1_, *p*_2_, ..., *p*_*T*_ satisfies *p*_1_=*x*_*i*,_*p*_*T*_=*x*_*j*_ and the density of *p*_*t*+1_ goes directly to *p*_*t*_ for any *x*_*i*_ and *x*_*j*_, then the density of *x*_*j*_ can reach *x*_*i*_. This finding indicated that the density can achieve transitivity. Transmitted samples *p*_1_, *p*_2_, ..., *p*_*T*−1_ in the sequence are all core objects because only the core object can direct the density of other samples.
5. Density connection: if both samples *x*_*i*_ and *x*_*j*_ can achieve the density of *x*_*k*_ due to the core object sample *x*_*k*_, then *x*_*i*_ density connection is called *x*_*j*_.

The specific process of DBSCAN clustering analysis is presented as follows:

1. Input: use sample set *D*=(*x*_1_, *x*_2_, ..., *x*_*m*_) and neighborhood parameter (*ε*, MinPts) in the measurement method of sample distance.
2. Initialize the core object set Ω=∅, cluster number *k*=0, and unaccessed sample set Γ=*D* and divide clusters into *C*=∅.
3. The *ε*− neighborhood subsample set *N*_*ε*_(*x*_*j*_) of sample *x*_*j*_ is obtained using distance measurement. If the number of samples in the subsample set meets|*N*_*ε*_(*x*_*j*_)| ≥ MinPts, then sample *x*_*j*_ will be added to the core object sample set Ω=Ω ∪ {*x*_*j*_} at this time.
4. If the core object set is Ω=∅, then the algorithm ends; otherwise, proceed to Step (5).
5. Randomly select a core object *o* in the core object set Ω to initialize the current cluster core object queue Ω_*cur*_={*o*}, class number *k*=*k*+1, and current cluster sample set *C*_*k*_={*o*} and then update the unaccessed sample set Γ=Γ − {*o*}.
6. If the current cluster core object queue is Ω_*cur*_=∅, then the current cluster cluster *C*_*k*_ is generated and cluster *C*={*C*_1_, *C*_2_, ..., *C*_*k*_} and core object set Ω=Ω − *C*_*k*_are updated. Go to Step (4); otherwise, update the core object set Ω=Ω − *C*_*k*_.
7. The current cluster core object queue Ω_*cur*_selects a core object *o*′and determines all *ε*− neighborhood subsample sets *N*_*ε*_(*o*′) through the neighborhood distance threshold *ε*. Let Δ=*N*_*ε*_(*o*′)∩Γ, update the current cluster sample set *C*_*k*_=*C*_*k*_ ∪ Δ, unvisited sample set Γ=Γ − Δ, and Ω_*cur*_=Ω_*cur*_ ∪ (Δ∩Ω) − *o*′; and proceed to Step (6).
8. The output result is the cluster partition *C*={*C*_1_, *C*_2_, ..., *C*_*k*_}.

The core idea of the DBSCAN clustering algorithm is to start from a core point and gradually expand to the density-accessible region to obtain an area where any two points are identically connected, and this area contains core and boundary points.

## 4. Problem Description and Model Building

With the continuous development of cold chain logistics industry, customer demand will change with the influence of season, personal taste, product quality, and other factors. Now most of the path optimization problems, the inventory is generally able to meet customer needs, but in the real distribution process, there will also be inventory cannot meet customer needs. In this case, it affects the total cost and distribution route planning, so two scenarios of out-of-stock and no-out-of-stock are designed in this study. Cold chain distribution enterprises implement differentiated management on customers in the process of distribution and adopt hard time window service for Class A customers with high priority and soft time window service for other customers in the no-out-of-stock scenario. Cold chain goods are allocated according to customer priority from high to low and hard time window is adopted for Class A customers with high priority in the case of out-of-stock.

### 4.1. Basic Assumptions of the Model

In order to build a vehicle routing optimization model with mixed time windows, the following assumptions are set according to the literature [9, 13, 14]:

1. A distribution center and multiple customers are distributed in different areas of the same city.
2. The cold chain distribution center has several identical refrigerated distribution vehicles, which are in good condition and presents a maximum load capacity greater than or equal to the cold chain demand of a single customer.
3. Refrigerated trucks run according to predesigned distribution routes and serve certain customers without midway assignment phenomenon.
4. Loading and unloading efficiency in the process of loading and unloading is the same as the unit loading and unloading cost.
5. The influence of customer priority on the distribution route and that of shortage of the distribution center on the distribution route in the process of distribution are considered.
6. Unit freight is the same and the influence of traffic congestion, refrigerated vehicle failure, and external risks on the speed of refrigerated vehicle is ignored in the process of distribution.

### 4.2. Establishment of the Cold Chain Distribution Model

The cold chain distribution center shall deliver cold chain goods to *n* customers and provide cold chain distribution services to customers within the specified time window of each customer.

#### 4.2.1. Model Parameters

Symbols of the urban cold chain vehicle route optimization model are listed in Table 1.

#### 4.2.2. Analysis of Cost Variables

*(1) Fixed Cost*. (*F*_1_) The fixed cost of the refrigerated vehicle traveling from the cold chain distribution center to the customer is positively correlated with the number of refrigerated vehicles. Driver salary, refrigerated vehicle depreciation, and maintenance costs are important components of fixed costs. Fixed costs can be expressed as follows:

(1) $$\begin{array}{c} F_{1}=\overset{C}{\underset{c=1}{\sum }}\overset{N}{\underset{j=1}{\sum }}x_{0j}^{c}f_{c}\text{,} \end{array}$$

where *x*_0*j*_^*c*^ is the decision variable when the refrigerated vehicle *c* is enabled in the distribution center; otherwise, *x*_0*j*_^*c*^ is 0.

*(2) Green Cost* (*F*_2_). The green cost includes fuel consumption and carbon emission costs of refrigerated vehicles. The oil consumption of refrigerated vehicles is solved via load estimation method. The oil consumption rate per unit distance is *η*_0_ when the refrigerated vehicle load is zero. The oil consumption rate per unit distance is *η*_*∗*_ when the refrigerated vehicle is fully loaded. A certain linear relationship exists between the load of the refrigerated vehicle and the fuel consumption rate. Hence, the fuel consumption per unit distance of normal driving when the load of the refrigerated vehicle is *M* is calculated as follows:

(2) $$\begin{array}{c} \eta \left(M\right)=\eta_{0}+\frac{\eta_{*}-\eta_{0}}{Q_{0}}M\text{.} \end{array}$$

The fuel consumption from customer *i* to customer *j* is calculated as follows:

(3) $$\begin{array}{c} v_{\text{oil}}=\eta \left(Q_{ij}\right)d_{ij}\text{,} \end{array}$$

where *η*(*Q*_*ij*_) refers to the fuel consumption of a refrigerated vehicle with load capacity of *Q*_*ij*_ that drives directly from customer *i* to customer *j* units of distance and *d*_*ij*_ for customers *i* to *j* distance. The fuel consumption in the entire delivery process when the refrigerated vehicle completes the delivery service to all customers is expressed as follows:

(4) $$\begin{array}{c} f_{\text{oil}}=p_{\text{oil}}\overset{C}{\underset{c=1}{\sum }}\overset{N}{\underset{i=0}{\sum }}\overset{N}{\underset{j=0}{\sum }}x_{ij}^{c}\eta \left(Q_{ij}\right)d_{ij}\text{,} \end{array}$$

where *p*_*oil*_ is the price of oil.

Ottmar [21] demonstrated that a linear relationship also exists between carbon emissions and oil consumption, that is, carbon emissions = oil consumption × CO_2_ emission coefficient. Environmental cost in the entire distribution process is expressed as follows:

(5) $$\begin{array}{c} f_{\text{tax}}=\tau \varpi f_{\text{oil}}\text{,} \end{array}$$

where *τ* is the carbon tax and *ϖ* is the carbon emission coefficient.

The green cost in the process of refrigerated vehicle running given that fuel consumption and carbon emission costs present a certain linear relationship with fuel consumption is expressed as follows:

(6) $$\begin{array}{c} F_{2}=f_{\text{oil}}+f_{\text{tax}}=p_{\text{oil}}\overset{C}{\underset{c=1}{\sum }}\overset{N}{\underset{i=0}{\sum }}\overset{N}{\underset{j=0}{\sum }}x_{ij}^{c}\eta \left(Q_{ij}\right)d_{ij}+\\ \tau \varpi \overset{C}{\underset{c=1}{\sum }}\overset{N}{\underset{i=0}{\sum }}\overset{N}{\underset{j=0}{\sum }}x_{ij}^{c}\eta \left(Q_{ij}\right)d_{ij}\text{.} \end{array}$$

*(3) Refrigeration Cost* (*F*_3_). The refrigeration cost refers to the cost of maintaining the temperature in the compartment of refrigerated vehicles. This cost is solved via the load estimation method. The refrigeration consumption rate per unit time is *δ*_0_ when the refrigerated vehicle is empty. The refrigeration consumption rate per unit time is *δ*_*∗*_ when the refrigerated vehicle is full. A certain linear relationship exists between the refrigeration cost and the refrigerated vehicle load. The refrigeration cost per unit time when the refrigerated vehicle is loaded with *M* is expressed as follows:

(7) $$\begin{array}{c} \delta \left(M\right)=\delta_{0}+\frac{\delta_{*}-\delta_{0}}{Q}M\text{.} \end{array}$$

Therefore, the refrigeration cost in the normal running process of refrigerated vehicles is expressed as follows:

(8) $$\begin{array}{c} F_{3}=\delta \left(Q_{ij}\right)\overset{C}{\underset{c=1}{\sum }}\overset{N}{\underset{i=0}{\sum }}\overset{N}{\underset{j=0}{\sum }}\left(t_{ij}^{c}x_{ij}^{c}\right.+T_{i}\left.y_{i}^{c}\right)\text{.} \end{array}$$

*(4) Cost of Cargo Damage* (*F*_4_). Cold chain goods are sensitive to changes in the external environment, and their deterioration costs are positively correlated with changes in time and temperature. Arrhenius equation is used in this study to calculate the deterioration cost of cold chain goods in the cold chain carriage due to the change in external ambient temperature. The Arrhenius equation is expressed as follows: *K*=*Ae*^−(*E*_*a*_/*RT*)^, where *E*_*a*_ is the reaction activation energy; *K* is the reaction rate constant, which is only affected by temperature; *A* is the frequency factor; *R* is the molar gas constant; and *T* is the absolute temperature. Assuming that the temperature in the refrigerated compartment remains unchanged, the deterioration rate of cold chain goods in the compartment remains the same but changes exponentially with the increase of time. Therefore, its metamorphism function is

(9) $$\begin{array}{c} G_{teu}\left(t\right)=G_{teu}\left(0\right)\times K\times e^{-u\left(t_{ij}^{c}x_{ij}^{c}+T_{i}y_{i}^{c}\right)}\text{,} \end{array}$$

where *G*_*teu*_(*t*) represents the quality of cold chain goods at time *t*; *G*_*teu*_(0) represents the quality of cold chain goods when intact, that is, the quality of cold chain goods at time 0; *K* is the change constant of cold chain goods at a certain constant temperature; *μ* represents the sensitivity of cold chain goods to time, also referred to as sensitivity coefficient, such that high sensitivity of cold chain goods to time corresponds to a small sensitivity coefficient and vice versa. *μ* can be expressed as follows:

(10) $$\begin{array}{c} \mu =K_{\max}\exp\left(-\frac{E_{a}}{RT}\right)\text{.} \end{array}$$

Therefore, the cargo damage cost of cold chain goods during transportation is expressed as follows:

(11) $$\begin{array}{c} F_{4}=p*Q_{ij}\overset{C}{\underset{c=1}{\sum }}\overset{N}{\underset{i=0}{\sum }}\overset{N}{\underset{j=0}{\sum }}\left[1-e^{-u\left(t_{ij}^{c}x_{ij}^{c}+T_{i}y_{i}^{c}\right)}\right]. \end{array}$$

*(5) Penalty Costs* (*F*_5_). The customer satisfaction model is built according to supply chain theory, and the satisfaction is taken as the function of the customer receiving time *t*. The fuzzy number is a convex fuzzy set defined on the real number field. The commonly used fuzzy number functions are triangular fuzzy number and trapezoidal fuzzy number.

In this article, trapezoidal fuzzy number *t*_*k*_=(*e*_*k*_, *e*_*k*_′, *l*_*k*_′, *l*_*k*_) is used. The highest satisfaction of customer *Bi* in receiving cold chain goods within time window (*e*_*k*_′, *l*_*k*_′) is (*l*_*k*_′, *l*_*k*_) and the penalty cost is 0 at this time. Customer *Bi* receives cold chain goods within (*e*_*k*_, *e*_*k*_′) or 3. Although the time of receiving goods is not within the expected time window of customer *Bi*, the further the time of receiving goods deviates from the expected time window, the lower the satisfaction of customer *Bi* will be. At this time, the penalty cost gradually increases as time goes forward or backward. If customer *Bi* receives the cold chain goods outside the time window (*e*_*k*_, *l*_*k*_), then customer *Bi* will not accept the goods. In this case, the satisfaction is quantified as 0, the penalty cost is a maximum real number Ε, and according to Liu et al. [22], the target model has no solution. Therefore, the time for customer *Bi* to receive supplies *t*_*k*_ is expressed by the corresponding penalty function *ρ*(*t*_*k*_) as follows:

(12) $$\begin{array}{c} \rho \left(t_{k}\right)=\left\{\begin{array}{c} Ε,t_{k}<e_{k,}\\ \gamma_{1}\left(t_{k}-e_{k}\right),e_{k}\leq t_{k}<e_{k,}^{\prime }\\ 0,e_{k}^{\prime }\leq t_{k}\leq l_{k}^{\prime }\text{,}\\ \gamma_{2}\left(l_{k}-t_{k}\right),l_{k}^{\prime }<t_{k}\leq l_{k,}\\ Ε,t_{k}>l_{k.} \end{array}\right. \end{array}$$

According to equation (12), the penalty cost can be shown in equation (13):

(13) $$\begin{array}{c} F_{5}=\overset{C}{\underset{c=1}{\sum }}\overset{N}{\underset{i=1}{\sum }}\rho \left(t_{i}\right). \end{array}$$

#### 4.2.3. Optimization Model of Urban Cold Chain Transport Route

The sum of fixed, refrigeration, green, cargo damage, and penalty costs is obtained in this study and used as the objective function to build an optimization model for the driving route of cold chain vehicles as follows:

(14) $$\begin{array}{c} \min\;F=F_{1}+F_{2}+F_{3}+F_{4}+F_{5}=\overset{C}{\underset{c=1}{\sum }}\overset{N}{\underset{j=1}{\sum }}x_{0j}^{c}f_{c}+\left(p_{oil}+\tau \varpi \right)\overset{C}{\underset{c=1}{\sum }}\overset{N}{\underset{i=0}{\sum }}\overset{N}{\underset{j=0}{\sum }}x_{ij}^{c}\eta \left(Q_{ij}\right)d_{ij}+\\ \delta \left(Q_{ij}\right)\overset{C}{\underset{c=1}{\sum }}\overset{N}{\underset{i=0}{\sum }}\overset{N}{\underset{j=0}{\sum }}\left(t_{ij}^{c}x_{ij}^{c}\right.+T_{i}\left.y_{i}^{c}\right)+p*Q_{ij}\overset{C}{\underset{c=1}{\sum }}\overset{N}{\underset{i=0}{\sum }}\overset{N}{\underset{j=0}{\sum }}\left[1-e^{-\mu \left(t_{ij}^{c}x_{ij}^{c}+T_{i}y_{i}^{c}\right)}\right]+\overset{C}{\underset{c=1}{\sum }}\overset{N}{\underset{i=1}{\sum }}\rho \left(t_{i}\right). \end{array}$$

s.t.

(15) $$\begin{array}{c} \overset{N}{\underset{i=1}{\sum }}q_{i}y_{i}^{c}\leq Q_{c},\forall c\text{,} \end{array}$$

(16) $$\begin{array}{c} \overset{C}{\underset{c=1}{\sum }}\overset{N}{\underset{i=1}{\sum }}\overset{N}{\underset{j=1}{\sum }}x_{ij}^{k}\leq r\text{,} \end{array}$$

(17) $$\begin{array}{c} \overset{C}{\underset{c=1}{\sum }}\overset{N}{\underset{i=1}{\sum }}\overset{N}{\underset{j=1}{\sum }}x_{ij}^{c}d_{ij}^{c}\leq D_{0}\text{,} \end{array}$$

(18) $$\begin{array}{c} \overset{C}{\underset{c=1}{\sum }}y_{i}^{c}=1,\forall i\text{,} \end{array}$$

(19) $$\begin{array}{c} \overset{C}{\underset{c=1}{\sum }}\overset{N}{\underset{j=0}{\sum }}x_{0j}^{c}=\overset{C}{\underset{c=1}{\sum }}\overset{N}{\underset{j=0}{\sum }}x_{j0}^{c}, \end{array}$$

(20) $$\begin{array}{c} \overset{N}{\underset{i=0}{\sum }}x_{ij}^{c}=y_{i}^{c},\forall j,c\text{,} \end{array}$$

(21) $$\begin{array}{c} \overset{N}{\underset{j=0}{\sum }}x_{ij}^{c}=y_{i}^{c},\forall i,c\text{,} \end{array}$$

(22) $$\begin{array}{c} \overset{C}{\underset{c=1}{\sum }}\overset{N}{\underset{i=1}{\sum }}\overset{N}{\underset{j=1}{\sum }}x_{ij-1}^{c}=\overset{C}{\underset{c=1}{\sum }}\overset{N}{\underset{i=1}{\sum }}\overset{N}{\underset{j=1}{\sum }}x_{ij+1}^{c}, \end{array}$$

(23) $$\begin{array}{c} \left\{\begin{array}{c} Q_{ij}=0,\left(q_{0}\leq \max Q_{ij}\right)\cup \min\left|q_{0}-Q_{ij}\right|,j=1,2,\cdots n\text{,}\\ \overset{j+x}{\underset{j=1}{\sum }}Q_{ij}=0,\left(q_{0}>\;\max Q_{ij}\right)\cup \min\left|q_{0}-\overset{j+x}{\underset{j=1}{\sum }}Q_{ij}\right|,x=1,2,\cdots ,n-1\text{,} \end{array}\right. \end{array}$$

(24) $$\begin{array}{c} \left\{\begin{array}{c} Q_{ih}=0,\left(q_{0}\leq \max Q_{ih}\right)\cup \min\left|q_{0}-Q_{ih}\right|,h=30-a-b,\cdots ,n\text{,}\\ \overset{h+y}{\underset{h=1}{\sum }}Q_{ih}=0,\left(q_{0}>\;\max Q_{ih}\right)\cup \min\left|q_{0}-\overset{h+y}{\underset{h}{\sum }}Q_{ih}\right|,y=1,2,\cdots h-1\text{,} \end{array}\right. \end{array}$$

(25) $$\begin{array}{c} t_{j}=t_{i}+T_{i}+t_{ij},\forall i,j\text{,} \end{array}$$

(26) $$\begin{array}{c} x_{ij}^{c}=\left\{\begin{array}{c} 1,\text{Refrrigeated truck }c\text{ drives directly from Customer }i\text{ to Customer }j\text{,}\\ 0,\text{otherwise,} \end{array}\right. \end{array}$$

(27) $$\begin{array}{c} y_{i}^{c}=\left\{\begin{array}{c} 1,\text{Refrigerated truck }c\text{ completes the distribution task of Customer }i\text{ ,}\\ 0,\text{otherwise.} \end{array}\right. \end{array}$$

Equation (15) indicates that the load capacity of the refrigerated vehicle is within its maximum approved load capacity. Equation (16) shows that the number of refrigerated vehicles required for this distribution task is within the number of vehicles planned by the company. Equation (17) presents that the total distance traveled by a refrigerated vehicle *c* is within the permitted upper limit *D*_0_. Equation (18) demonstrates that each customer is only visited once. Equation (19) denotes that the refrigerated vehicle starts from the distribution center to complete the distribution task and then returns to the distribution center. Equation (20) signifies that any customer is only allowed to set out once for refrigerated vehicles. Equation (21) describes that any customer only allows the refrigerated vehicle to arrive once. Equation (22) expresses that each customer has only one previous successor node and one subsequent node connected to it. Equation (23) exhibits that the distribution center selects and assesses customers according to the serial number and does not serve one or more customers close to the quantity of out-of-stock when the distribution center is out-of-stock while ignoring the priority of customers. Equation (24) in the distribution center out-of-stock situation, considering customer priorities, distribution center according to the customer priority order to the customer, reverse, distribution center is not close to the shortage of the service number one or a few customers, where A is the number of Class A customers and B is the number of Class B customers. Equation (25) shows the continuity of distribution. Equation (26) presents the decision variable of the route (*i*, *j*) of the refrigerated vehicle *c* equal to 1 when the refrigerated vehicle *c* directly travels from Customer *i* to Customer *j*; otherwise, the decision variable is equal to 0. Equation (27) is the decision variable equal to 1 when the refrigerated vehicle *c* provides delivery service for customer *i* and meets the delivery demand of customer *i*; otherwise, the decision variable is equal to 0.

## 5. Design Optimization Algorithm

The cold chain logistics distribution route problem with mixed time window and the cold chain distribution route problem considering the priority of customers are both proved to be NP-hard problem. In this article, an improved genetic algorithm is designed to solve the model to achieve a balance between the convergence speed and accuracy of the algorithm.

### 5.1. Chromosome Coding

**Figure 1:**
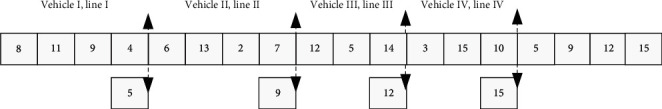
Example of chromosome coding.

Chromosome coding is the most important factor affecting the efficiency and result of genetic algorithm. Scientific and reasonable chromosome coding can improve the efficiency and result precision. The current study assumes one distribution center and *n* customers in the optimization problem of cold chain logistics distribution route. Chromosomes are constructed by using the coding method of randomly sorting customers. The end of the chromosome is the cumulative ordinal number of the customers that provide distribution service for a vehicle. The chromosome code is 1,2, ⋯, *n*+1, where 1 represents a distribution center and the rest are customers. Take Figure 1 as an example, where the number of customers is 2 to 15, (1,8,11,9,4,1), (1,6,13,2,7,1), (1,12,5,14,1), and (1,3,15,10,1) represent the four subroutes of cold chain distribution. Add the end of the chromosome as a set of solutions according to the sequence of vehicle driving routes and the cumulative ordinal number of customers in each subroute of (4,8,11,14). When considering customer priority for vehicle driving route optimization, Class A customers with high customer priority are set as hard time windows, whereas other customers are set as soft time windows. Randomly generated initial feasible solution *P*(0), starting from the distribution center search, choose from the distribution center is the nearest customer as the next access nodes, then the selected node as the current node selection from the current node is the nearest customer as the next access nodes, and so on when does not satisfy the constraint conditions of vehicle return cold chain distribution center. Repeat until all customers have been visited, and repeat the process until all initial populations have been generated.

### 5.2. Select Fitness Function

According to the survival of the fittest standard, the stronger the ability of the chromosome to adapt to the environment, the better the chromosome, and the higher the fitness value is at this time. The objective function of the model established in this article is minimum total cost *C*; hence, the fitness of the improved genetic algorithm is fitness=1/*C*.

### 5.3. Selection Operation

The basic idea of the roulette method is that the greater the probability of an individual choosing, the greater the fitness value. The specific steps are as follows:

(1) *C* represents the fitness function value of the initial individual, and *C*_*s*_ represents the sum of the fitness function values of all individuals in the population. The formula is as follows:
  (28) $$\begin{array}{c} C_{s}=\overset{n}{\underset{i=1}{\sum }}C\left(x_{i}\right),i=1,2,\cdots ,n\text{.} \end{array}$$
(2) Generate a random value in [0, *F*_*s*_].
(3) Solve the sum of the fitness function values of all individuals. When the sum exceeds *F*_*s*_, the last accumulated individual solution will be the selected individual. Additionally, the optimal individual solution is recorded to compare the optimal individual solution with the global optimal solution. If the optimal individual solution is better, then the global optimal solution is updated.

### 5.4. Cross Operation

Greedy algorithm is adopted to improve the crossover mutation operator, and the improved mutation rate is dynamic. The crossover probability is defined as:

(29) $$\begin{array}{c} \rho =\frac{1}{1+\exp\;\left(\kappa_{1}\Delta \right)}\text{,} \end{array}$$

where *κ*_1_ is a constant coefficient, Δ=fitness_avg_ − fitness_max_. fitness_avg_ is the average fitness value and fitness_max_ is the maximum fitness value. Create a random number *ϑ*, and *ϑ* ∈ [0,1]. If *ϑ* < *ρ*, select the given gene for crossover. Two groups of offspring are randomly generated as follows:

(30) $$\begin{array}{c} v_{1}=\\ \left(a_{1},{\;a}_{2},\cdots ,a_{m},{\;a}_{pos1},a_{pos1+1},\cdots ,{\;a}_{pos1+m},{\;a}_{pos2},{\;a}_{pos2+1},\cdots ,{\;a}_{pos2+m},\cdots ,a_{posm},{\;a}_{posm+1},\cdots ,{\;a}_{posm+m},{\;a}_{m}\right)v_{2}\\ =\left(b_{1},{\;b}_{2},\cdots ,b_{m},b_{pos1},{\;b}_{pos1+1},\cdots ,b_{pos1+m},{\;b}_{pos2},b_{pos2+1},\cdots ,{\;b}_{pos2+m},\cdots ,b_{posm},b_{posm+1},\cdots ,b_{posm+m},b_{m}\right)\text{.} \end{array}$$

Among them, *a*_1_, *a*_2_, ⋯, *a*_*m*_ is the second chromosome of the progeny of the 1,2, ⋯, *m* group. *a*_*pos*1_, *a*_*pos*1+1_, ⋯, *a*_*pos*1+*m*_ is the 1,2, ⋯, *m* chromosome of the mutation in the progeny of the first group. *b*_*pos*1_, *b*_*pos*1+1_, ⋯, *b*_*pos*1+*m*_ is the 1,2, ⋯, *m* chromosome of mutation in the second group of progeny, and so on.

### 5.5. Variation Operation

According to the characteristics of direct arrangement structure chromosome coding, the mutation operation of the parent chromosome is carried out by using the exchange mutation operator. Randomly select a paternal chromosome and randomly select a location on this chromosome to cut the DNA for single-point crossover. If a feasible solution can be generated through the mutation operation, then the random exchange mutation operator succeeds in mutation, and the mutant chromosomes are classified into their offspring to form a new population. Select, cross, and mutate the new population, and repeat the operation. Finally, the individual fitness function value and the group average fitness function value continue to increase. When the optimal individual fitness function value reaches a limit or the group average fitness function value and the optimal individual fitness function value no longer increase, the algorithm convergence ends.

## 6. Analysis of Calculation Examples

Data provided by a cold chain logistics company in Xiamen Free Trade Zone, which is an enterprise engaged in the distribution of imported fruits and provides cold chain fruit distribution services for customers, are used to verify the effectiveness of the algorithm. The central warehouse of the cold chain enterprise is considered the distribution center.

### 6.1. Instance-Related Data

Data were collected from 30 customers served by the logistics company. A1 is the distribution center with coordinates of (45,50) and B2∼B31 are customer points, and customer types are divided into four categories: strategic partner type (SPT), partner type (PT), parent company type (PCT), and ordinary type (OT). The improved cold chain distribution route optimization model of genetic algorithm is used to solve the problem. Values of 30 customer coordinates, demand, trapezoidal fuzzy time window, and priority evaluation indexes are listed in Table 2.

The approved load of refrigerated vehicle is set to 30 *t* and the unit running cost of vehicle is 10 yuan/km in the route optimization study. The speed of the vehicle is 60 km/h, and the cost of the vehicle is 100 yuan/vehicle. Parameters of the algorithm are set as follows: initial population number: 200, number of iterations: 1000, crossover probability: 0.8, and mutation probability: 0.1. Relevant parameters of the model are listed in Table 3.

### 6.2. Analysis of Clustering Results

**Figure 2:**
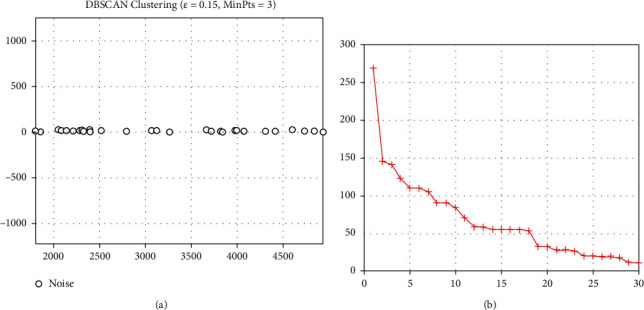
Results of DBSCAN cluster analysis of customer priority.

The DBSCAN cluster analysis model was solved according to the customer priority evaluation information in Table 2. The results of the DBSCAN cluster analysis of customer priority are shown in Figure 2. Customer priority is divided into three categories of A, B, and C in this study. The clustering results with three categories are shown in Table 4.

According to the Pareto law, A-type customers are key customers primarily managed by cold chain distribution enterprises. Therefore, expending a considerable amount of financial and material resources is necessary to improve the satisfaction of A-type customers. Cold chain distribution enterprises must communicate regularly with B-type customers to strengthen, maintain, and improve their relationship. Cold chain distribution enterprises are not required to manage C-type customers or expend a considerable amount of manpower, material, and financial resources on them. Classified management and control of customers should be carried out according to different priorities of customers, especially when the inventory is insufficient. The delivery service should be selected according to the priority of customers. Cold chain logistics enterprises carry out A, B, and C classification management schemes on customers and focus on key A-type customers to reduce blindness in work, improve the distribution efficiency of cold chain distribution centers, and enhance customer satisfaction and loyalty. The results of route optimization and analysis in Section 5.3 demonstrated that cold chain distribution enterprises adopt hard time window management for Class A customers with high customer priority to improve their satisfaction and maintain them but apply soft time window management for Type B and C customers when considering the optimization of cold chain logistics transportation route with customer priority.

### 6.3. Route Optimization Results and Analysis

Two scenarios are designed in this study to prove the effectiveness of the model. The cold chain logistics distribution route problem is solved without considering customer priority and considering customer priority in the case of no shortage in Scenario 1. The problem of cold chain logistics distribution route without considering customer priority and considering customer priority is solved in the situation of shortage in Scenario 2.

#### 6.3.1. No Shortage Scenario

**Figure 3:**
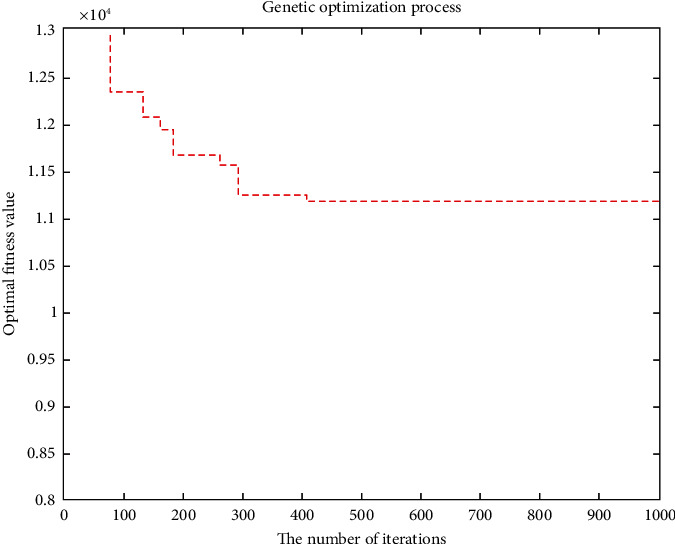
Convergence curve of the objective function.

**Figure 4:**
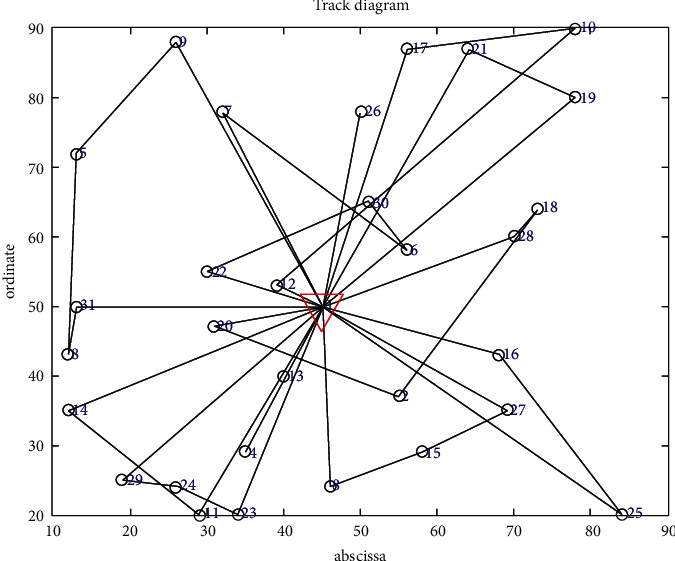
Optimal trajectory of the vehicle.

The improved genetic algorithm is used to solve the problem of no shortage, and the objective function is applied to minimize the cost without considering customer priority. The minimum total cost is RMB 11171.4216, and the total mileage is 1030.264 km. Eleven refrigerated cars were needed, and the algorithm took 100.3899 seconds. The convergence curve of the objective function and the optimal trajectory of the refrigerated vehicle are illustrated in Figures 3 and 4, respectively.

**Figure 5:**
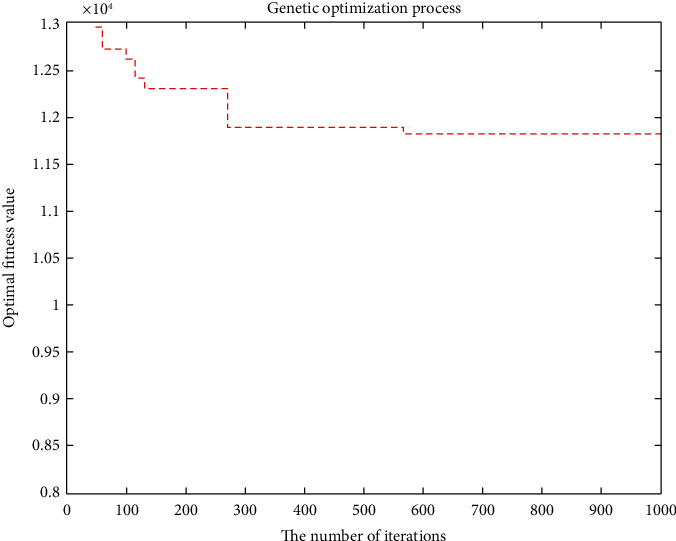
Convergence curve of the objective function.

**Figure 6:**
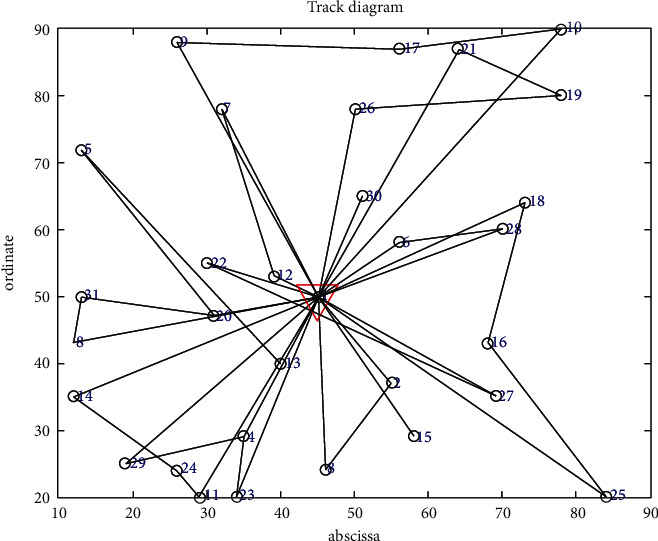
Optimal trajectory of the vehicle.

The objective function aims to minimize the cost considering customer priority in the case of no shortage. The minimum total cost is RMB 11,828.3755, and the total mileage is 1066.0542 km. Eleven refrigerated cars were needed, and the time was 105.2741 seconds. The convergence curve of the objective function and the optimal trajectory of the refrigerated vehicle are illustrated in Figures 5 and 6, respectively.

The comparison of the optimization results of the cold chain distribution route without considering customer priority and considering customer priority in the non-out-of-stock scenario is shown in Table 4.

#### 6.3.2. Out-of-Stock Situation

**Figure 7:**
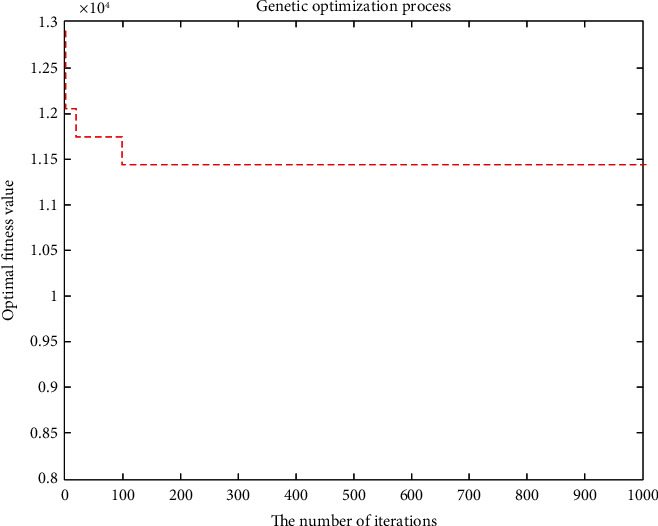
Convergence curve of the objective function.

**Figure 8:**
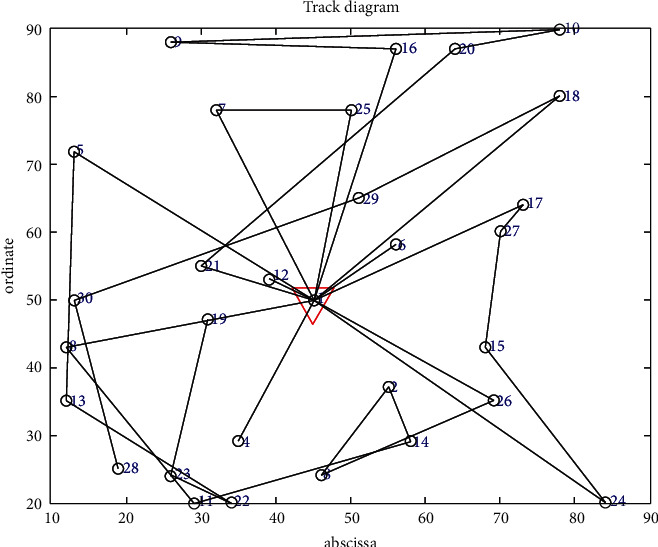
Optimal trajectory of the vehicle.

Assuming that the quantity of out-of-stock in a distribution center is 5 tons in a day, the improved genetic algorithm is adopted to solve the situation of out-of-stock and the objective function is applied to minimize the cost without considering customer priority. The minimum total cost is RMB 11446.7693, and the total mileage is 990.8133 km. Nine refrigerated vehicles are needed, and customer B13 is absent in the distribution service. The coordinate is (40,40), and the time is 110.0057 seconds. The convergence curve of the objective function and the optimal trajectory of the refrigerated vehicle are shown in Figures 7 and 8, respectively.

**Figure 9:**
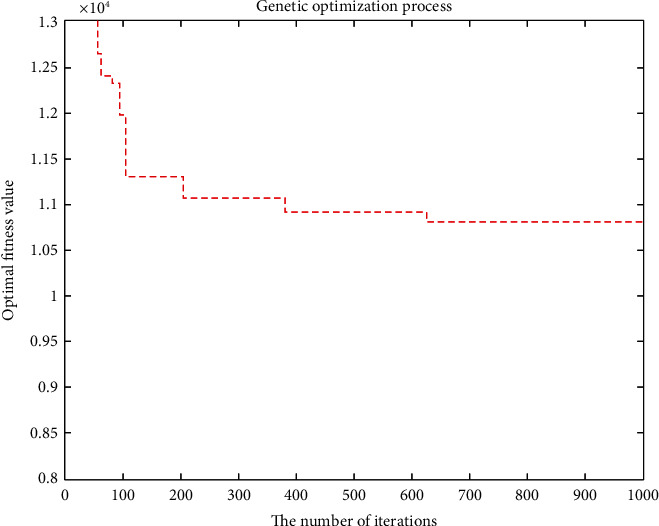
Convergence curve of the objective function.

**Figure 10:**
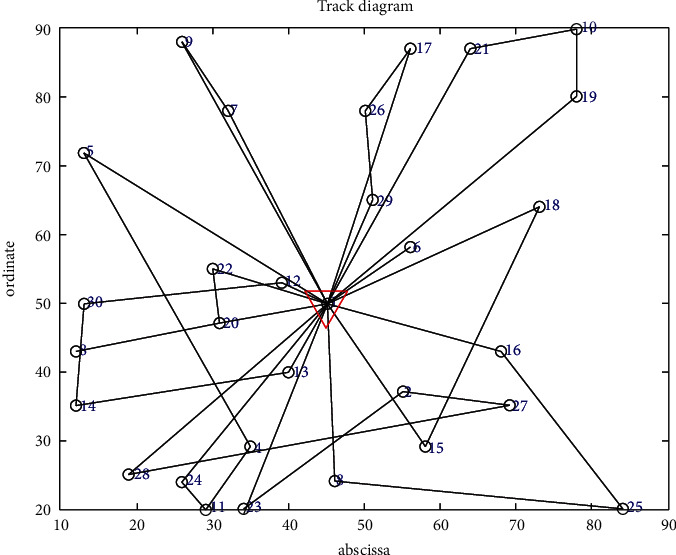
Optimal trajectory of the vehicle.

The objective function is applied to minimize the cost considering customer priority in the case of out-of-stock. The minimum total cost is RMB 10780.7221, and the total mileage is 971.6001 km. Ten refrigerated vehicles are needed, and delivery service is unavailable for customer B28. The coordinate is (70,60), and the time is 112.148323 seconds. The convergence curve of the objective function and the optimal route diagram of the refrigerated vehicle are presented in Figures 9 and 10, respectively.

The comparison between the optimization results of cold chain distribution route without considering customer priority and considering customer priority in the out-of-stock scenario is shown in Table 5.

### 6.4. Analysis of Experimental Results

Table 5 shows that the distribution cost and distance traveled by refrigerated vehicles are nearly the same as those of the optimized cold chain distribution route considering customer priority when the cold chain distribution route optimization that ignores customer priority requires 11 vehicles to complete the distribution task for 30 customers in the case of no shortage. Although the distribution cost increases by 656.954 and the total driving distance increases by 35.790, the satisfaction of key customers and the service level of the cold chain distribution center improve when customer priority is considered.  Table 6 presents that the distribution center needs to send 9 vehicles to complete the distribution task for 30 customers in the optimization of cold chain distribution route without considering customer priority and the distribution cost and distance of refrigerated vehicles is RMB 11,446.769 without customer B13 (40,40) for goods distribution in the case of stock shortage. However, the distribution center needs to send 10 vehicles to complete the distribution service without customer B28 (70,60) for delivery service and with a total cost of RMB 10780.722 in the optimization of cold chain distribution route considering customer priority. Although the refrigerated vehicle increases by 1 compared with the distribution plan that ignores the priority of customers, the distribution cost reduces by RMB 666.047. Therefore, the integrated distribution scheme considering customer priority in Tables 5 and 6 meets not only the needs of key customers but also improves the service level of key customers without significantly increasing the cost. The empirical analysis achieves the expected results, and the distribution scheme that considers customer priority is consistent with requirements of market economy and customer satisfaction.

### 6.5. Algorithm Performance Analysis

**Figure 11:**
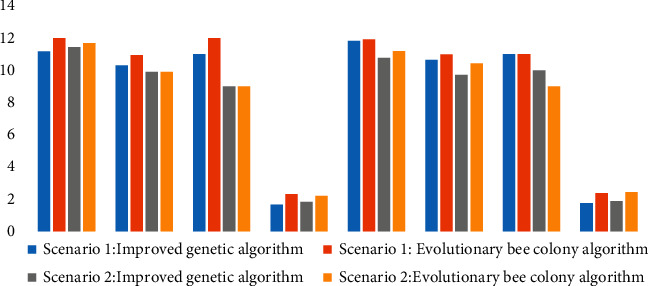
Performance comparison between improved genetic algorithm and evolutionary bee colony algorithm.

To verify the validity of the algorithm, this article also writes the evolutionary bee colony algorithm in the same hardware environment, using the above example data, using the same parameter settings, under the two scenarios, the two algorithms in distribution costs, vehicle total mileage, vehicle number, and algorithm running time.  Figure 11 shows the comparison of the results of the two algorithms.

In the two scenarios, whether the priority of customers is considered or not, four groups of results are solved. In terms of the total cost, the total mileage of vehicles driven, and the number of vehicles required, the average target value of the improved heritage algorithm is better than that of the evolutionary bee colony algorithm. In terms of program running time, the average time of the improved genetic algorithm is 106.954 s, whereas that of the evolutionary bee colony algorithm is 139.718 s. The efficiency of the improved genetic algorithm is 23.451% higher than that of the evolutionary bee colony algorithm. Based on the above analysis, the improved genetic algorithm adjusts the crossover probability in the design of the greedy algorithm, according to the fitness function for dynamic adjustment, and speeds up the optimization. It can effectively avoid algorithm into local optimum and improve the efficiency of the algorithm search performance, to verify the effectiveness of the improved genetic algorithm.

## 7. Conclusion

The time window constraint of trapezoidal fuzzy membership function based on the analysis of characteristics of urban cold chain transportation is introduced in this study. The cold chain distribution route optimization is analyzed on the basis of whether the distribution center is out-of-stock and customer priority is considered. A cold chain distribution route optimization model considering customer priority was constructed, and two scenarios of not-out-of-stock and out-of-stock were solved using the improved genetic algorithm. The experimental results showed that the model can obtain the optimal distribution route with the minimum cost and adjust the cold chain distribution route according to the inventory of the distribution center to improve customer satisfaction and reduce the cold chain distribution cost and opportunity loss. The model and algorithm in this study can provide method support and reference for the scientific planning of transportation route of cold chain transportation enterprises. However, the distribution products considered in this article all have the same temperature requirements, and traffic and other emergencies during the distribution process are not considered. In future research, we can solve the fresh and cold chain distribution problem under the time-varying road network and heterogeneous vehicles combined with the specific problems in reality.

## Figures and Tables

**Table 1:** Notation for the model.

Notation	Definition
*N*	Customer assembly.
*C*	Refrigerated truck assembly.
*f*_oil_	Fuel cost consumed by refrigerated vehicles during distribution
*f*_*C*_	Fixed cost of refrigerated truck *c*
*q*_*i*_	Customer *i* cold chain product demand
*p*	Unit price of cold chain products transported by refrigerated trucks
*t*_*Ci*_	Arrival time of refrigerated truck *c* to customer *i*
*t*_*C*0_	Departure time of refrigerated truck *c* from the distribution center
*T*_*i*_	Refrigerated truck service hours for customer *i*
*Q*_*ij*_	Load of refrigerated truck when traveling from customer *i* to customer *j*
*Q*	Maximum inventory of cold chain distribution center
*Q*_0_	Maximum load of refrigerated vehicles
*q*_0_	Amount of supplies that are out-of-stock in the distribution center in the out-of-stock scenario
*γ*_1_	Penalty factor for refrigerated vehicles that deliver earlier than the upper limit of the customer's agreed time window
*γ*_2_	Penalty coefficient for refrigerated trucks that deliver later than the lower limit of the customer's agreed time window
*Q*_*lh*_	The needs of each customer are sorted by customer priority from high to low
*D*_0_	Total distance traveled by refrigerated trucks
*r*_0_	Total number of vehicles available

**Table 2:** Customer coordinate, demand, time window and priority evaluation index information.

Customer	*X*, *Y* (km)	Demand for medical materials (ton)	Time window (min)	Order amount (monthly average)	Order frequency (month)	Satisfaction	Loyalty	Customer type	Customer level
b2	55, 37	2.44	(0, 10, 30, 40)	2321.79	14	0.789	0.756	SPT	D
b3	46, 24	4.96	(0, 15, 50, 70)	4936.23	2	0.878	0.603	SPT	A
b4	35, 29	3.84	(0, 20, 40, 60)	3815.41	15	0.838	0.725	PT	A
b5	13, 72	9.10	(0, 10, 20, 30)	4598.36	30	0.903	0.648	PCT	D
b6	56, 58	6.31	(0, 30, 60, 90)	3721.8	10	0.719	0.512	PT	B
b7	32, 78	1.72	(0, 40, 90, 130)	3975.79	16	0.775	0.53	SPT	C
b8	12, 43	5.09	(0, 10, 20, 30)	4306.23	13	0.745	0.694	PCT	A
b9	26, 88	10.33	(0, 15, 45, 60)	4078.16	13	0.807	0.534	SPT	A
b10	78, 90	2.53	(0, 10, 50, 60)	4742.25	9	0.615	0.896	SPT	C
b11	29, 20	4.64	(0, 10, 30, 40)	2385.29	14	0.705	0.727	PCT	E
b12	39, 53	7.11	(0, 30, 60, 70)	4416.13	9	0.739	0.607	PCT	D
b13	40, 40	5.00	(0, 5, 15, 20)	3994.67	16	0.666	0.711	PCT	D
b14	12, 35	1.69	(0, 20, 60, 90)	1801.32	10	0.94	0.711	PCT	A
b15	58, 29	3.00	(0, 10, 20, 30)	3121.29	22	0.793	0.557	SPT	B
b16	68, 43	3.21	(0, 10, 30, 40)	3840.97	5	0.815	0.815	PT	E
b17	56, 87	4.06	(0, 40, 80, 120)	2387.15	25	0.614	0.906	PT	E
b18	73, 64	7.82	(0, 15, 30, 45)	2787.81	9	0.807	0.639	PT	B
b19	78, 80	9.66	(0, 60, 90, 120)	2306.4	27	0.633	0.748	OT	A
b20	31, 47	2.02	(0, 15, 45, 60)	2051.06	24	0.628	0.919	OT	A
b21	64, 87	5.31	(0, 10, 60, 70)	2211.73	10	0.613	0.732	PCT	C
b22	30, 55	7.50	(0, 25, 55, 80)	2281.68	20	0.928	0.672	SPT	B
b23	34, 20	6.00	(0, 20, 40, 60)	3261.75	7	0.741	0.817	PT	B
b24	26, 24	10.89	(0, 5, 20, 30)	2083.18	23	0.662	0.7	OT	C
b25	84, 20	4.55	(0, 20, 60, 80)	4847	15	0.871	0.797	SPT	A
b26	50, 78	1.36	(0, 10, 30, 45)	3066.95	23	0.647	0.606	SPT	D
b27	69, 35	5.94	(0, 5, 25, 40)	2135.94	21	0.656	0.63	PT	E
b28	70, 60	6.20	(0, 25, 55, 80)	2397.97	4	0.924	0.802	OT	D
b29	19, 25	4.33	(0, 15, 45, 60)	3665.45	26	0.899	0.643	PT	B
b30	51, 65	6.50	(0, 20, 40, 60)	1855.45	1	0.679	0.797	PT	D
b31	13, 50	2.44	(0, 35, 70, 105)	2518.96	22	0.669	0.517	OT	B

**Table 3:** Related parameter setting.

*v*	*p*	*f*_*c*_	*Q*_0_	*r*_0_	*Q*	*τ*	*ϖ*	*T*_0_	*E*_*a*_	*p*_oil_	*D*_0_	*δ*	*δ*_*∗*_	*η*	*η*_*∗*_	*γ*_1_	*γ*_2_	*K*	*R*

60 km/h	18 yuan/kg	100	30 t	15	300 t	30	2.669 kg/l	2°C	100 kJ/mol	5.56 yuan/l	100 km	0	30	0.18	0.41	100 yuan/h	300 yuan/h	5*∗*10^14^	8.314 J/mol/k

**Table 4:** Clustering results when the number of categories is 3.

Customer	Sort Kdist	Percentage	Cumulative percentage	Cumulative percent -age of quantity	Priority level
b18	269.1809	0.1352	0.1352	0.0333	A
b5	145.4148	0.0731	0.2083	0.0666	
b23	141.2589	0.0710	0.2792	0.1000	
b31	122.3427	0.0615	0.3407	0.1333	
b8	109.9955	0.0553	0.3960	0.1666	
b12	109.9955	0.0553	0.4512	0.2000	
b10	104.9840	0.0527	0.5040	0.2333	

b3	90.2166	0.0453	0.5493	0.2666	B
b25	90.2166	0.0453	0.5946	0.3000	
b9	83.5681	0.0420	0.6366	0.3333	
b21	70.6973	0.0355	0.6721	0.3666	
b6	58.6206	0.0294	0.7016	0.4000	
b29	58.6206	0.0294	0.7310	0.4333	
b14	54.9193	0.0276	0.7586	0.4666	
b30	54.9193	0.0276	0.7862	0.5000	
b15	54.3678	0.0273	0.8135	0.5333	
b26	54.3678	0.0273	0.8408	0.5666	
b27	52.8926	0.0266	0.8674	0.6000	
b20	32.1519	0.0162	0.8835	0.6333	
b24	32.1519	0.0162	0.8997	0.6666	
b4	27.5195	0.0138	0.9135	0.7000	
b16	27.5195	0.0138	0.9273	0.7333	
b22	25.7132	0.0129	0.9403	0.7666	

b2	20.4665	0.0103	0.9505	0.8000	C
b19	20.4665	0.0103	0.9608	0.8333	
b7	19.1180	0.0096	0.9704	0.8666	
b13	19.1180	0.0096	0.9800	0.9000	
b28	16.3045	0.0082	0.9882	0.9333	
b11	11.7260	0.0059	0.9941	0.9666	
b17	11.7260	0.0059	1.0000	1.0000	

**Table 5:** No shortage comparison of results.

Vehicle	Vehicle routing without considering customer priority	Vehicle	Vehicle routing considering customer priority
1	a1 ⟶ b9 ⟶ b5 ⟶ b8 ⟶ b31 ⟶ a1	1	a1 ⟶ b13 ⟶ b5 ⟶ b20 ⟶ b31 ⟶ b8 ⟶ a1
2	a1 ⟶ b4 ⟶ b13 ⟶ a1	2	a1 ⟶ b2 ⟶ b3 ⟶ a1
3	a1 ⟶ b28 ⟶ b18 ⟶ b2 ⟶ b20 ⟶ a1	3	a1 ⟶ b26 ⟶ b19 ⟶ b21 ⟶ a1
4	a1 ⟶ b7 ⟶ b6 ⟶ b30 ⟶ b22 ⟶ a1	4	a1 ⟶ b25 ⟶ b16 ⟶ b18 ⟶ a1
5	a1 ⟶ b27 ⟶ b15 ⟶ b3 ⟶ a1	5	a1 ⟶ b7 ⟶ b12 ⟶ a1
6	a1 ⟶ b17 ⟶ b10 ⟶ b12 ⟶ a1	6	a1 ⟶ b15 ⟶ a1
7	a1 ⟶ b26 ⟶ a1	7	a1 ⟶ b11 ⟶ b24 ⟶ b14 ⟶ a1
8	a1 ⟶ b25 ⟶ b16 ⟶ a1	8	a1 ⟶ b29 ⟶ b4 ⟶ b23 ⟶ b30 ⟶ b1
9	a1 ⟶ b29 ⟶ b24 ⟶ b23 ⟶ a1	9	a1 ⟶ b9 ⟶ b17 ⟶ b10 ⟶ a1
10	a1 ⟶ b21 ⟶ b19 ⟶ a1	10	a1 ⟶ b6 ⟶ b28 ⟶ a1
11	a1 ⟶ b14 ⟶ b11 ⟶ a1	11	a1 ⟶ b22 ⟶ b27 ⟶ a1

**Table 6:** Out-of-stock comparison of results.

Vehicle	Vehicle routing without considering customer priority	Vehicle	Vehicle routing considering customer priority
1	a1 ⟶ b4 ⟶ a1	1	a1 ⟶ b9 ⟶ b7 ⟶ a1
2	a1 ⟶ b8 ⟶ b11 ⟶ b14 ⟶ b2 ⟶ b3 ⟶ b26 ⟶ a1	2	a1 ⟶ b19 ⟶ b10 ⟶ b21 ⟶ a1
3	a1 ⟶ b28 ⟶ b30 ⟶ b29 ⟶ b18 ⟶ a1	3	a1 ⟶ b13 ⟶ b14 ⟶ b30 ⟶ b12 ⟶ a1
4	a1 ⟶ b17 ⟶ b27 ⟶ b15 ⟶ b24 ⟶ a1	4	a1 ⟶ b3 ⟶ b25 ⟶ b16 ⟶ a1
5	a1 ⟶ b5 ⟶ b13 ⟶ b22 ⟶ b23 ⟶ b19 ⟶ a1	5	a1 ⟶ b6 ⟶ a1
6	a1 ⟶ b16 ⟶ b9 ⟶ b10 ⟶ b20 ⟶ b21 ⟶ a1	6	a1 ⟶ b18 ⟶ b15 ⟶ a1
7	a1 ⟶ b7 ⟶ b25 ⟶ a1	7	a1 ⟶ b23 ⟶ b2 ⟶ b27 ⟶ b28 ⟶ a1
8	a1 ⟶ b12 ⟶ a1	8	a1 ⟶ b29 ⟶ b26 ⟶ b17 ⟶ a1
9	a1 ⟶ b6 ⟶ a1	9	a1 ⟶ b24 ⟶ b11 ⟶ b4 ⟶ b5 ⟶ a1
		10	a1 ⟶ b22 ⟶ b20 ⟶ b8 ⟶ a1

## Data Availability

The data used to support the findings of this study can be obtained from the corresponding author upon request.
